# Cardiovascular Metrics Associated With Prevention of Aging-Related Parkinsonian Signs Following Exercise Intervention in Sedentary Older Rats

**DOI:** 10.3389/fnagi.2021.775355

**Published:** 2021-12-15

**Authors:** Ella A. Kasanga, Joel Little, Tamara R. McInnis, Nicoleta Bugnariu, J. Thomas Cunningham, Michael F. Salvatore

**Affiliations:** ^1^Department of Pharmacology and Neuroscience, University of North Texas Health Science Center, Fort Worth, TX, United States; ^2^Department of Physiology and Anatomy, University of North Texas Health Science Center, Fort Worth, TX, United States; ^3^School of Health Sciences, University of the Pacific, Sacramento, CA, United States

**Keywords:** exercise, parkinsonism, aging, heart rate, predictive validity

## Abstract

Preservation of motor capabilities is vital to maintaining independent daily living throughout a person's lifespan and may mitigate aging-related parkinsonism, a progressive and prevalent motor impairment. Physically active lifestyles can mitigate aging-related motor impairment. However, the metrics of physical activity necessary for mitigating parkinsonian signs are not established. Consistent moderate intensity (~10 m/min) treadmill exercise can reverse aging-related parkinsonian signs by 20 weeks in a 2-week on, 2-week off, regimen in previously sedentary advanced middle-aged rats. In this study, we initiated treadmill exercise in sedentary 18-month-old male rats to address two questions: (1) if a rest period not longer than 1-week off exercise, with 15 exercise sessions per month, could attenuate parkinsonian signs within 2 months after exercise initiation, and the associated impact on heart rate (HR) and mean arterial pressure (MAP) and (2) if continuation of this regimen, up to 20 weeks, will be associated with continual prevention of parkinsonian signs. The intensity and frequency of treadmill exercise attenuated aging-related parkinsonian signs by 8 weeks and were maintained till 23 months old. The exercise regimen increased HR by 25% above baseline and gradually reduced pre-intervention MAP. Together, these studies indicate that a practicable frequency and intensity of exercise reduces parkinsonian sign severity commensurate with a modest increase in HR after exercise. These cardiovascular changes provide a baseline of metrics, easily measured in humans, for predictive validity that practicable exercise intensity and schedule can be initiated in previously sedentary older adults to delay the onset of aging-related parkinsonian signs.

## Introduction

After age 60, the risk of motor disability increases substantially (Bennet et al., 1996; Avendano et al., 2009; Wahrendorf et al., 2013). These disabilities can manifest as mild parkinsonian signs such as bradykinesia, gait, and/or balance disturbances (Bennet et al., 1996; Buchman et al., 2016; Oveisgharan et al., 2021), affecting 15% by age 65 and nearly 50% by age 80 (Katzman and Terry, 1992; Bennet et al., 1996; Buchman et al., 2016; Oveisgharan et al., 2021). Previously, this progressive locomotor impairment (Buchman et al., 2016) was considered to be a common benign consequence of aging (Katzman and Terry, 1992). However, it is now recognized that aging-related parkinsonism is progressive, like Parkinson's disease (PD), and associated with adverse health outcomes, including increased risk of disability (Fleischman et al., 2007), mild cognitive impairment, cognitive decline (Richards et al., 1993; Louis et al., 2010; Buchman et al., 2011), loss of independent living, and death (Bennet et al., 1996; Fleischman et al., 2007; Oveisgharan et al., 2021). Deficits in nigrostriatal function contribute to parkinsonian signs in human aging (Buchman et al., 2011, 2012), with postmortem neuropathology revealing evidence of nigral neuronal loss (Fearnley and Lees, 1991; Ross et al., 2004; Buchman et al., 2012). Consistent with human aging, rat and non-human primate models of aging show loss of tyrosine hydroxylase or dopamine in the substantia nigra in association with parkinsonian signs (Emborg et al., 1998; Yurek et al., 1998; Gerhardt et al., 2002; Salvatore et al., 2009). Moreover, experimental reduction of dopamine in substantia nigra in young rats produces parkinsonian signs (Salvatore et al., 2019), and the increases therein in old rats alleviate parkinsonian signs (Pruett and Salvatore, 2013). Unfortunately, no current therapies exist to mitigate parkinsonian signs in the elderly without PD, as the central nervous system (CNS) mechanisms of motor decline have not been thoroughly elucidated (Sorond et al., 2015; Clark et al., 2019).

Given the prevalence of parkinsonian signs and an increasing number of individuals over age 60, identifying effective non-pharmacological interventions to slow the progression of aging-related parkinsonism is vital (Wahrendorf et al., 2013; Varma et al., 2016). There is evidence that physical activity levels influence the risk of motor impairments with aging (Hillsdon et al., 2005; Denison et al., 2013), including parkinsonism (Buchman et al., 2018; Santos et al., 2018; Oveisgharan et al., 2020). A more active lifestyle in the elderly population may reduce the risk of parkinsonism (Buchman et al., 2018; Santos et al., 2018). This evidence strongly suggests the neurobiological mechanisms engaged by a physically active lifestyle (with regular exercise) offset CNS mechanisms that produce parkinsonism (Santos et al., 2018). Identifying exercise-responsive mechanisms that counter parkinsonian signs, currently an unmet need, can reveal relevant targets for preventing or mitigating parkinsonism severity.

Rodent models of aging can evaluate exercise impact on CNS mechanisms and may be of high translational value (Arnold and Salvatore, 2014, 2016; Arnold et al., 2017). However, such studies must be tethered into a regimen of exercise intensity, duration, and frequency that is realistic in humans, particularly those who have had sedentary lifestyles (Neufer et al., 2015; Brossia-Root et al., 2016; Zeiss et al., 2017) or people of advanced age. We previously reported that moderate-intensity, foot-shock-free, treadmill exercise regimen produced a reversal of parkinsonian signs in previously sedentary 18-month-old Brown-Norway Fischer 344 F_1_ hybrid (BNF) rats (Arnold and Salvatore, 2014; Arnold et al., 2017). This regimen also affected markers of nigrostriatal integrity (Arnold and Salvatore, 2016), lending support to a possible approach to interrogate CNS mechanisms of a physically active lifestyle that attenuate parkinsonism with advancing age. Notably, the antiparkinsonian effects of the aforementioned study were mitigated by 5 months, with a consistent exercise/rest regimen of ~2 consecutive weeks on and 2 consecutive weeks off exercise (Arnold et al., 2017).

To optimally translate the results of exercise impact in older rats into a possible antiparkinsonian benefit to the human condition, this study had two primary goals. First, we determined if the same moderate-intensity exercise regimen could mitigate parkinsonian signs in a shorter time than 5 months, by reducing the number of maximum consecutive rest days between exercise days by half (Arnold et al., 2017) but keeping the number of exercise days per month the same. In this study, two rest days were interspersed between three consecutive five exercise days per week, followed by 1-week rest, the last week of each month, during which locomotor assessments were made. For predictive validity of this regimen to alleviate parkinsonian signs in older sedentary humans, we evaluated cardiovascular indices of heart rate (HR) and mean arterial pressure (MAP) (Wisløff et al., 2001; Høydal et al., 2007) immediately before and after exercise and longitudinally for 8 weeks. In an additional longitudinal study of exercise impact, we determined if the continual application of this exercise regimen could mitigate parkinsonian signs till 23–24 months old, thus representing an exercise intervention at an approximate human age range equivalent of 50–75 years old (Spangler et al., 1994; Quinn, 2005).

## Materials and Methods

### Animals

Male BNF rats aged 18 months old (*n* = 39, 393–500 g) were obtained from aging rodent colonies under the auspices of the National Institute on Aging (Charles River). This strain is established in aging studies, with a well-established decline in locomotor activity akin to aging-related parkinsonism particularly bradykinesia/hypokinesia with reduced ambulatory counts/time and distance traveled starting ~3 months prior to reaching 18 months old (Spangler et al., 1994; Salvatore et al., 2009, 2017; Arnold et al., 2017). Rats were singly housed under controlled colony conditions and fed *ad libitum*, with a 12-h reverse light-dark cycle (lights on at 20:00 h), ensuring that both exercise and locomotor assessments occurred during the awake hours.

Two different studies were conducted. In Study I, 18-month-old male BNF rats (*n* = 19) were used to determine the impact of a 2-month duration exercise regimen on cardiovascular measures (HR, MAP, and maximal oxygen consumption, VO_2_max), and locomotor function. In Study II, another cohort of 18-month-old male BNF rats (*n* = 20) was used to determine the impact of the same treadmill exercise regimen on locomotor function for 5 months duration. Rats were acclimated to the vivarium for a minimum of 1 week prior to experiments. Reporting of *in vivo* experiments (ARRIVE) guidelines were adhered to, and all procedures were approved by the animal care and use committee at the UNT Health Science Center at Fort Worth.

### Treadmill Exercise Regimen

**Figure 1:**
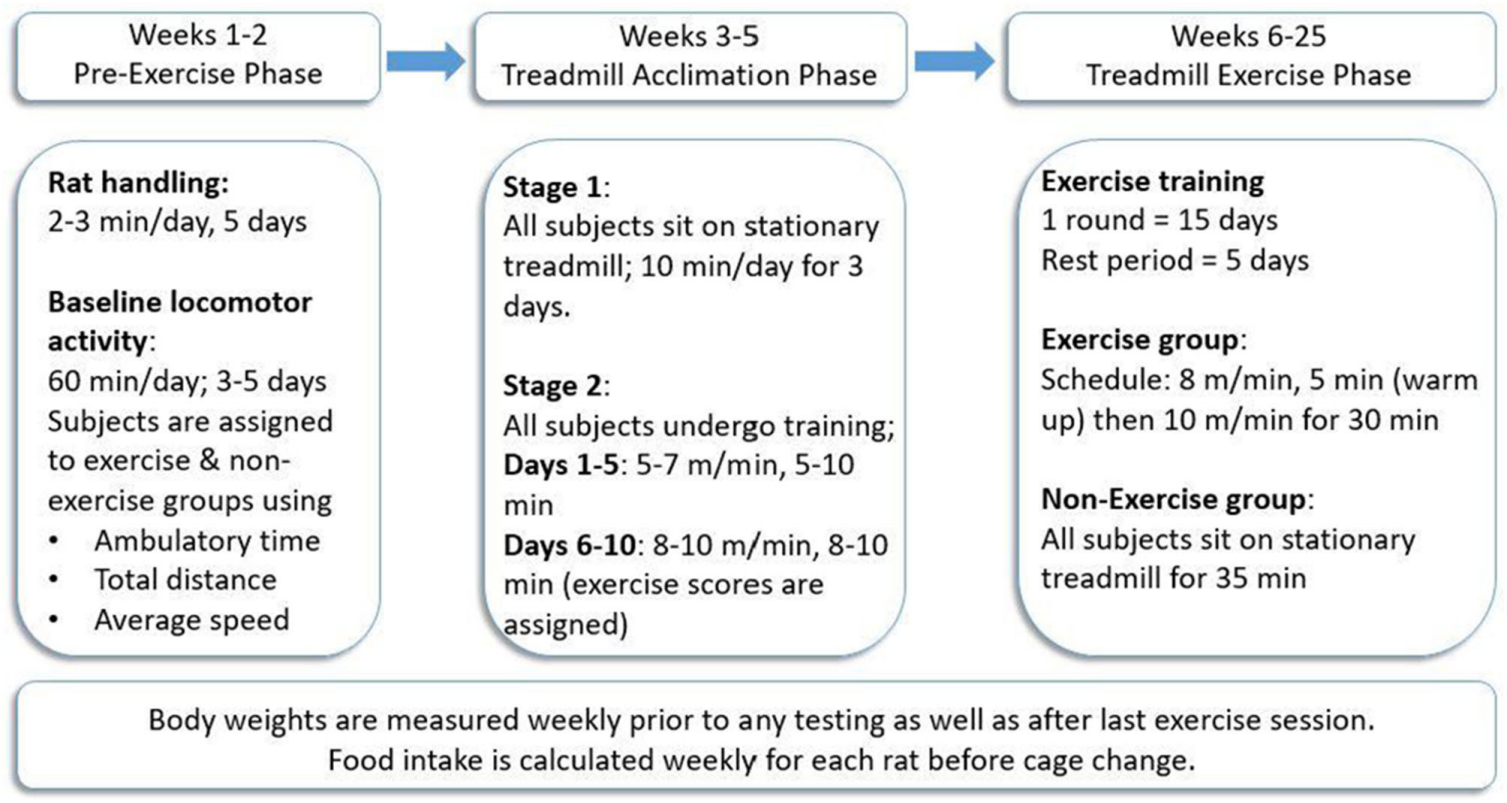
Treadmill exercise regimen. All rats (*n* = 39) were trained to exercise on the treadmill in the acclimation phase after initial locomotor testing to determine and establish baseline locomotor performance for each rat. The exercise was conducted 5 days/week for three consecutive weeks, followed by 5 days of rest wherein locomotor testing was conducted either for the first 3 days (Study I) or on all 5 days (Study II). This regimen was continued for 2 months (Study I) or 5 months (Study II). Procedure follows modification of exercise regimen (Arnold and Salvatore, 2014).

In both studies, a foot-shock-free exercise regimen was employed as previously described (Arnold and Salvatore, 2014, 2016; Arnold et al., 2017). To maximize compliance to treadmill exercise, this exercise regimen was divided into three main phases: pre-exercise phase, treadmill acclimation phase, and treadmill exercise phase (Figure 1), following our previously established protocol (Arnold and Salvatore, 2014) (Refer to Supplementary Figure 1 for a visual of treadmill environment using the BNF rat).

### Pre-exercise Phase

This consisted of 1 week of rat handling followed by 3–5 days of baseline locomotor activity testing (described below).

### Treadmill Acclimation Phase

All treadmill acclimation and exercise sessions were conducted on foot-shock-free treadmills (Exer 3/6 Treadmill, S/N 140282 Add. 220, Columbus Instruments Inc., Columbus, OH). All rats (later assigned to non-exercise (NE) or exercise (EX) groups) underwent the treadmill acclimation phase to ensure each subject demonstrated the capacity to exercise. The acclimation phase was divided into two main stages. In stage 1, all rats were placed on a stationary treadmill for 10 min/day for three consecutive days. In stage 2, which is further divided into two main phases, acclimation 1 (first 5 days) and acclimation 2 (next 5 days), all rats trained on a moving treadmill for 10 days. The speed of and duration on the treadmill were gradually increased until rats ran at 10 m/min for 10 min on the last day of training. During the last week of acclimation, exercise compliance scores, ranging from 0 to 4, were assigned to each rat to verify compliance to the speed and duration of the session without assistance from the experimenter. An exercise score of 4 was assigned for rats that completed the session without any assistance, whereas a score of 0 denoted non-compliance to the regimen, and the test subject did not complete the session. A score of 3 indicated the subject did not require assistance for 75% of the total session time, and a score of 2 was assigned when test subjects required experimenter assistance for 50% of the session (Arnold and Salvatore, 2014, 2016; Arnold et al., 2017). After completion of acclimation, rats were assigned to the EX or NE groups such that there was no significant difference in baseline locomotor activity or exercise compliance scores.

### Treadmill Exercise Phase

Exercise sessions were conducted in monthly rounds comprised of 15 total days of exercise (five consecutive days of exercise followed by 2 days of rest each week for three consecutive weeks). In the fourth week of the study, no exercise was conducted and locomotor activity was assessed for the first 3 days (Study I) or 5 days (Study II) following exercise. Rats in the EX group were exercised at a speed of 8 m/min for 5 min (warm-up) and then 10 m/min for 30 min. Rats in the NE group were placed on a stationary treadmill for 35 min at the same frequency as the EX group throughout both studies.

### Locomotor Assessment

Spontaneous locomotor activity was monitored in a darkened room during the rat awake cycle using automated activity chambers (VersaMax Animal Activity Monitoring System, Columbus Instruments, Inc., Columbus, OH). Activity monitors consist of a 41 × 41 × 31 cm plexiglass box with a grid of infrared beams to automatically record activity. Locomotor activity was evaluated in movement parameters defined as the ambulatory time (AT, time of initiated movements), total distance (DT, cm), and speed (cm/s). To account for daily inherent variance in locomotor activity within each test subject, as previously established for this strain (Spangler et al., 1994; Salvatore et al., 2009), the locomotor assessment was conducted for 1 h daily for 3 days in Study I or 5 days in Study II. Unlike other studies from our group (Salvatore et al., 2009; Arnold et al., 2017), we found evidence for habituation in Study II within the five sessions at each monthly assessment (data not shown). We, therefore, used the mean of the final four daily assessments to statistically evaluate the respective motor parameters at each monthly assessment. Prior to beginning exercise in either study, baseline locomotor activity was established before exercise initiation to ensure no significant difference in baseline locomotor activity in rats assigned to the EX or NE groups. Locomotor activity was assessed every 4 weeks in both studies, beginning 3 days after completing each third consecutive week of exercise.

## Study I: Impact of Treadmill Exercise on Cardiovascular Function

### Radiotelemetry Transmitter Implantation

After acclimation to the vivarium, rats underwent surgery to implant telemetry devices (Abdominal Aorta Implant, Data Sciences International (DSI model S10, St. Paul, MN, USA) to enable recording of HR and MAP. Under 2% isoflurane inhalation anesthesia and aseptic conditions, rats received implants of an abdominal aortic catheter attached to a CA11PA-C40 radiotelemetry transmitter (Musch et al., 1989). The renal artery (above the iliac bifurcation) was tied with 2–0 silk to temporarily occlude blood flow. A 23-gauge needle was used to introduce the blood pressure telemetry transmitter catheter (~2 cm). Vetbond secured the catheter to the aorta. The transmitter body was sutured in the peritoneal cavity on the muscle wall with 4–0 prolene sutures. The 3–0 vicryl antimicrobial sutures were used to close the muscle followed by the skin. Rats were monitored closely, recovered for 1 week, underwent treadmill acclimation, and then grouped thereafter into the EX (*n* = 10) or NE groups (*n* = 9).

### Collection of Cardiovascular Data

Baseline HR and MAP readings were measured during phases 1 and 2 of the acclimation phase for 30 min using DSI telemetry receivers and MX2 matrix using DSI Ponemah Acquisition Software. Each rat was contained in its home cage and placed onto a receiver to record MAP and HR data. Measurements were taken at one-min intervals for 5 min immediately prior to, and immediately after, exercise, both in the home cage of the rat. These measures were collected on each first and fifth (last) day of each week of exercise and averaged. For off exercise weeks (4th week and 8th week in study), one to three evaluations were taken at the start of the week following exercise, and the first measure was used in statistical analysis. Mean MAP and HR were calculated from the 5-min assessments and evaluated statistically as mean of the first 2 min or 5 min.

## Study II: Impact of Longitudinal Exercise on Aging-Related Motor Decline

Rats underwent treadmill acclimation and were grouped into the EX (*n* = 10) and NE (*n* = 10) groups after which rats exercised on the regimen described above for 5 months. To evaluate the impact of aging and exercise, the weight of rats was tracked weekly on the first day of exercise or locomotor testing. Food intake was also tracked weekly (prior to cage change).

### Statistics

All results were analyzed using GraphPad Prism 8 (La Jolla, CA, USA) with *p*-values ≤0.05 considered as significant.

To determine the influence of the exercise regimen on body weight and locomotor parameters, a two-way repeated-measures ANOVA was used, comparing the measures of each subject in the NE and EX groups. For each test subject, the percentage of baseline value was used in the ANOVA analyses for each movement parameter. The effect of exercise, age, and interaction between exercise and age were assessed. With evidence of statistical significance, *post-hoc* measures at each time point were done with an unpaired *t*-test.

To determine the influence of exercise on HR and MAP, a two-way repeated-measures ANOVA was used, comparing pre-intervention HR and MAP to the post-intervention measures. Impact of exercise on the baseline and pre-exercise/intervention HR and MAP over the course of the study also used two-way repeated-measures ANOVA (sphericity was assumed). The effect of the session, weeks of regimen, and interaction between session and weeks of the regimen were assessed for significance. To compare the EX and NE groups on pre- and post-intervention HR and MAP at specific time points, an unpaired *t*-test was used. To determine the impact of exercise on VO_2_ max, an unpaired *t*-test was used to compare the NE vs. EX groups. The Grubb's test was used to identify outliers at each assessment, with outlier statistic set at α = 0.05.

In Study I, 80% of the exercise group had compliance scores of ≥50%, whereas 20% (two rats) had compliance scores <50%. Results from these two rats were excluded from statistical analysis. In Study II, 70% of the exercise group had compliance scores of ≥50%, whereas 30% (three rats) had compliance scores <50%. Results from these three rats were excluded from statistical analyses. The locomotor results were analyzed for each assessment period, and the Grubb's test identified outliers at each assessment, with outlier statistic set at α = 0.05.

## Results

### Exercise-Mediated Prevention of Aging-Related Parkinsonian Signs: Study I

**Figure 2:**
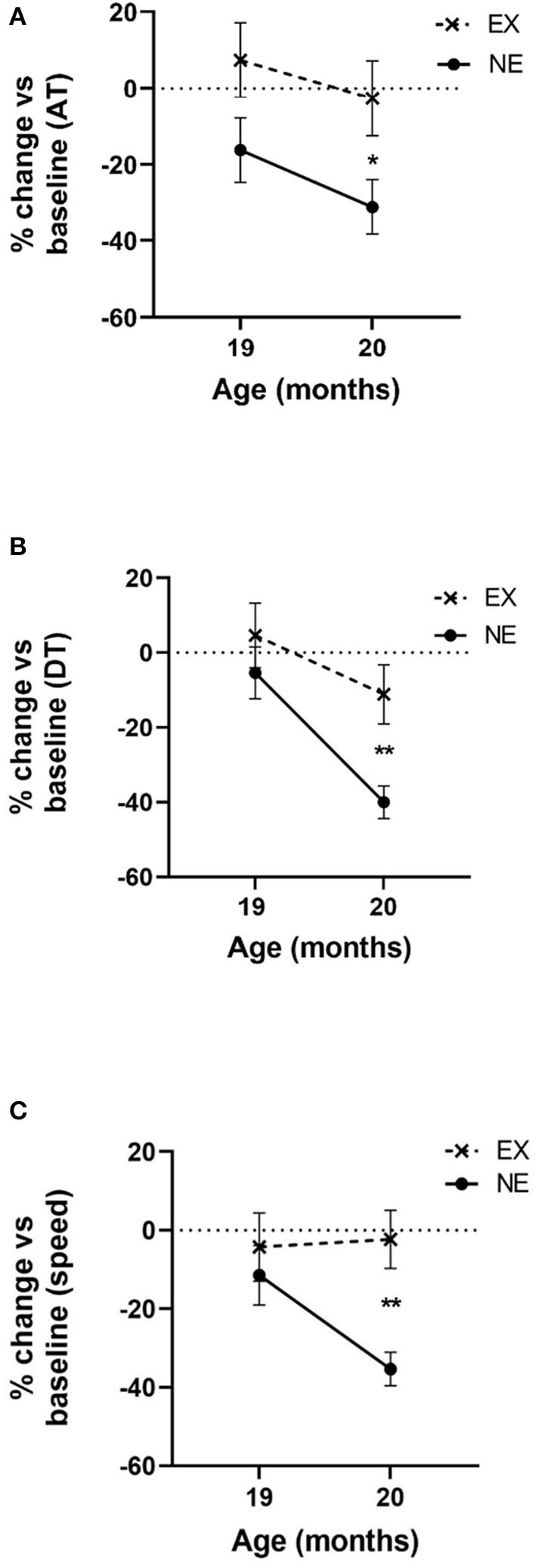
Impact of 2 months of exercise regimen (Study I) on locomotor parameters. Results represent the percentage of baseline values that was established at 18 months old and the mean of three 1 h evaluations in the open field for 19 and 20 months old. **(A)** Ambulatory time (AT). Movement initiation and frequency are represented as the mean AT. Two months of aging reduced AT [*F*_(1,13)_ = 6.25, *p* = 0.026]. Exercise (EX) attenuated aging-related decline in AT [*F*_(1,15)_ = 4.98, *p* = 0.041]. No interaction between exercise and age was observed [*F*_(1,13)_ = 0.16, ns]. At 20 months old, the decrease from baseline AT was significantly less in the EX group (*t* = 2.33, ^*^p = 0.035, df = 14). **(B)** Distance traveled (DT). Movement frequency and speed are represented as the mean DT. Two months of aging reduced DT [*F*_(1,13)_ = 19.66, *p* = 0.0007]. EX attenuated aging-related decline in DT [*F*_(1,15)_ = 5.32, *p* = 0.036]. No interaction between exercise and age on DT was observed [*F*_(1,13)_ = 2.37, ns]. At 20 months old, the decrease from baseline DT was significantly less in the EX group (*t* = 3.19, ^**^p = 0.007, df = 14). **(C)** Speed. Two months of aging reduced speed [*F*_(1,13)_ = 4.71, *p* = 0.049]. The EX group had a near significant effect on aging-related decline in speed [*F*_(1,15)_ = 3.96, *p* = 0.065] and near significant interaction between exercise and age [*F*_(1,13)_ = 4.38, *p* = 0.057]. At 20 months old, the decrease in speed from baseline was significantly less in the EX group (*t* = 4.01, ^**^p = 0.002, df = 13).

The significant aging-related decline in all three motor parameters (AT, DT, and speed) occurred at 20 months old in the NE group (Figures 2A–C). The monthly exercise regimen (3 weeks of 5 days exercise, 2 rest days per exercise week, and one full week rest) mitigated these aging-related parkinsonian-like impairments. Notably, this exercise/rest regimen mitigated these parkinsonian-like effects 3 months sooner than previously reported (Arnold et al., 2017), with the only key difference being that the longest rest period between exercise sessions was reduced from 2 to 1 week.

### Impact of Treadmill Exercise on Cardiovascular Functions

**Figure 3:**
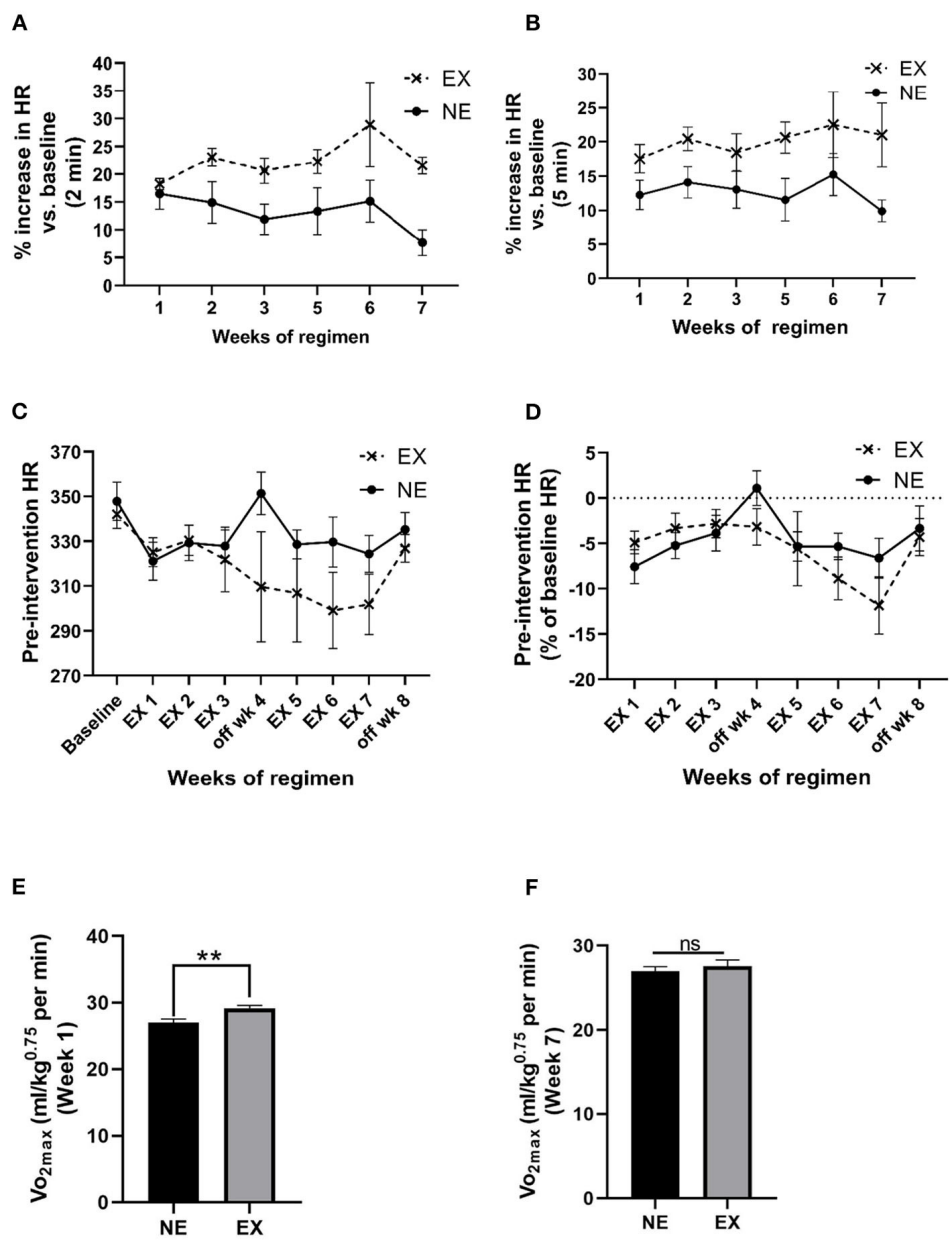
Impact of an exercise regimen on heart rate (HR) and calculated VO_2_max. **(A)** Treadmill exercise increases baseline HR (2 min mean). Mean HR increased the first 2 min immediately after the 35 min regimen, and to a greater extent in the EX vs. the NE group (placed on a stationary treadmill for 35 min). Exercise [*F*_(1,13)_ = 11.28, *p* = 0.005]. No significant interaction between exercise and weeks of exercise [*F*_(5,56)_ = 0.89, ns]. No significant effect of weeks of regimen [*F*_(5,56)_ = 1.11, ns]. **(B)** Treadmill exercise increases baseline HR (5 min mean). The percentage increase in HR in the EX group remained above baseline levels over 5 min immediately following exercise vs. the NE group. Exercise [*F*_(1,13)_ = 8.26, *p* = 0.013]. No significant interaction between exercise and weeks of exercise [*F*_(5,58)_ = 0.44, ns]. No significant effect of weeks of regimen [*F*_(5,58)_ = 0.67, ns]. **(C)** Exercise regimen impact on pre-intervention (resting) HR. Interaction of exercise with the duration of the exercise regimen on baseline HR was near significance [*F*_(8,104)_ = 1.85, *p* = 0.076], weeks of regimen had a significant effect on HR [*F*_(8,104)_ = 3.13, *p* = 0.003]. Exercise [*F*_(1,13)_ = 1.39, ns]. **(D)** Exercise regimen impact on pre-intervention HR (percentage of baseline HR). There was no significant interaction of exercise with the weeks of regimen [*F*_(7,86)_ = 1.26, ns]. Impact of weeks of regimen on HR as percentage baseline was significant [*F*_(7,86)_ = 3.37, *p* = 0.003]. The EX group was not different in percentage change in baseline HR across the study [*F*_(1,13)_ = 0.47, ns]. **(E)** Calculated VO_2_max post-week 1 of exercise. Analyses revealed a significant increase in the exercise after 1 week of exercise (*t* = 3.332, ^**^p < 0.01, df = 13). **(F)** Calculated VO_2_max post-week 7 of exercise. There was no significant difference between the two groups at the end of the exercise regimen (*t* = 0.701, ns, df = 13).

The mean HR, 2 min immediately after cessation of exercise, increased above baseline levels between 18% and 29% (overall mean ± SEM = 22.5% ± 1.4%) and was significantly greater compared with the NE group (Figure 3A; Supplementary Figure 2B). The mean increase was maintained for at least 5 min after exercise cessation (Figure 2B). Interestingly, after exposure to the stationary treadmill, HR also increased between 9% and 17% above baseline in the NE group vs. baseline levels (Supplementary Figure 2A).

We evaluated baseline HR obtained prior to initiation of the regimen against the mean pre-intervention HR (5 min mean, two times per week) for each week. Week of regimen significantly affected HR and the percentage of baseline over the course of the 8-week study, but no significant overall difference in the EX group against the NE group occurred for these measures (Figures 3C,D). There was no significant correlation between HR and locomotor performance at the study conclusion (Supplementary Table 1).

Maximal oxygen consumption during exercise was estimated based on the work of Høydal et al. (2007), calculated using the HR and the average rat VO_2_max at the treadmill speed, using the equation, *y* = 162*x*−1, where *x* is the speed of the treadmill in meters/second. After 1 week of exercise, there was ~9% increase in VO_2_max in the EX group (Figure 3E). After 6 weeks of exercise, no significant difference in VO_2_max was observed, denoting an adaptation in the EX group (Figure 3F).

**Figure 4:**
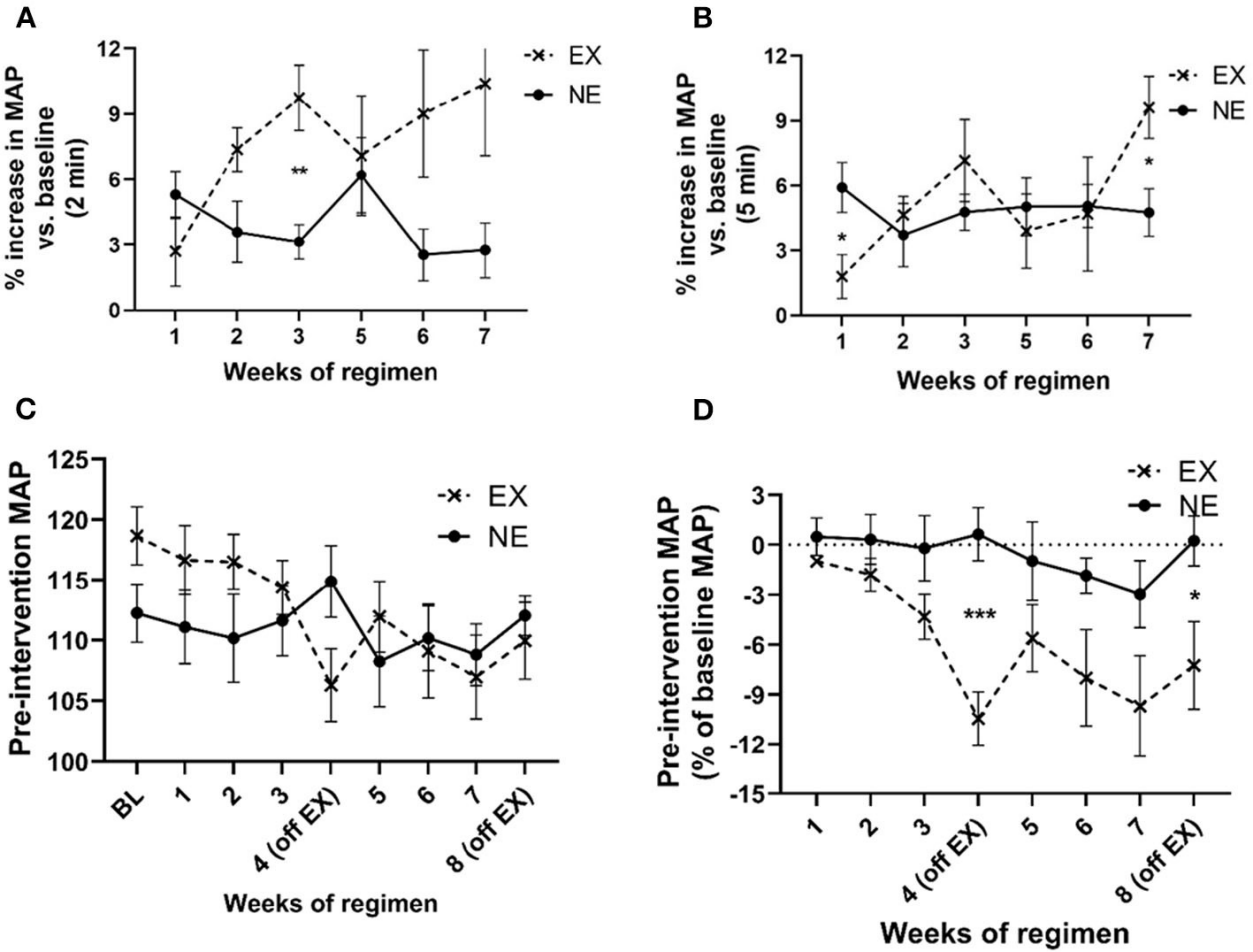
Impact of exercise regimen on mean arterial pressure (MAP). **(A)** Treadmill exercise increases baseline MAP (2 min mean). The mean MAP within the first 2 min immediately after the 35 min regimen increased to a greater extent in the EX vs. the NE group. There was a significant interaction between exercise and weeks of regimen [*F*_(5,59)_ = 2.63, *p* = 0.033]. Exercise [*F*_(1,13)_ = 4.98, *p* = 0.044], weeks of regimen [*F*_(5,59)_ = 0.94, ns]. Significant *post-hoc t*-test results during third week exercise (*t* = 4.10, ^**^p = 0.002, df = 11). **(B)** Treadmill exercise increases baseline MAP (5 min mean). The mean MAP in first 5 min immediately after the 35 min regimen was not significantly different between the EX and NE groups, although there was a significant interaction between exercise and weeks of regimen [*F*_(5,58)_ = 2.35, *p* = 0.05]. Exercise [*F*_(1,13)_ = 0.68, ns], weeks of regimen [*F*_(5,58)_ = 1.8, ns]. **(C)** Exercise regimen impact on pre-intervention (resting) MAP. There was a highly significant interaction of exercise with weeks of regimen on baseline MAP [*F*_(8,98)_ = 3.49, *p* = 0.001] mean of two 5 min evaluation each time point, per week), and significant effect on MAP as a function of weeks of regimen [*F*_(8,98)_ = 3.76, *p* = 0.0007]. Exercise [*F*_(1,13)_ = 0.01, ns]. **(D)** Exercise regimen impact on pre-intervention MAP (percent of baseline MAP). There was a significant interaction of exercise with the duration of exercise regimen [*F*_(7,81)_ = 2.28, *p* = 0.036], with an overall significant decrease (as percent of baseline (resting) MAP) established prior to initiation of the regimen, as a function of number of weeks of exercise [*F*_(7,81)_ = 3.67, *p* = 0.002]. There was a highly significant effect of exercise on baseline MAP {as percentage of baseline [*F*_(1,13)_ = 9.71, *p* = 0.008]}. Significant *post-hoc t*-test results; EX (off week 4: *t* = 4.86, ^***^p < 0.0005, df = 11); EX off week 8: *t* = 2.47, ^*^p < 0.05, df = 12).

MAP increased above baseline levels for 2 min immediately following exercise and to a greater extent than the NE group (Figure 4A). This increase in MAP diminished over 5 min immediately post-exercise, although significant interaction between weeks of regimen and exercise was still evident by week 7 (Figure 4B). The exercise regimen affected baseline MAP levels established prior to initiating exercise. There was a significant interaction between exercise and weeks of regimen on MAP and a significant effect of the duration (weeks) of the regimen (Figure 4C). Moreover, as a percentage of baseline (pre-regimen) MAP, exercise produced a highly significant decrease in baseline MAP that was established prior to starting the eight weeklong regimens, with significant differences in the EX group, duration of the regimen, and interaction between exercise and weeks of regimen (Figure 4D). As seen with HR, exposure to both the stationary and running treadmill significantly increased MAP above pre-exposure levels (Supplementary Figures 3A,B). Also, there was no significant correlation between MAP and locomotor performance at the study conclusion (Supplementary Table 2).

### Longitudinal Exercise Impact on Aging-Related Parkinsonian Signs

**Figure 5:**
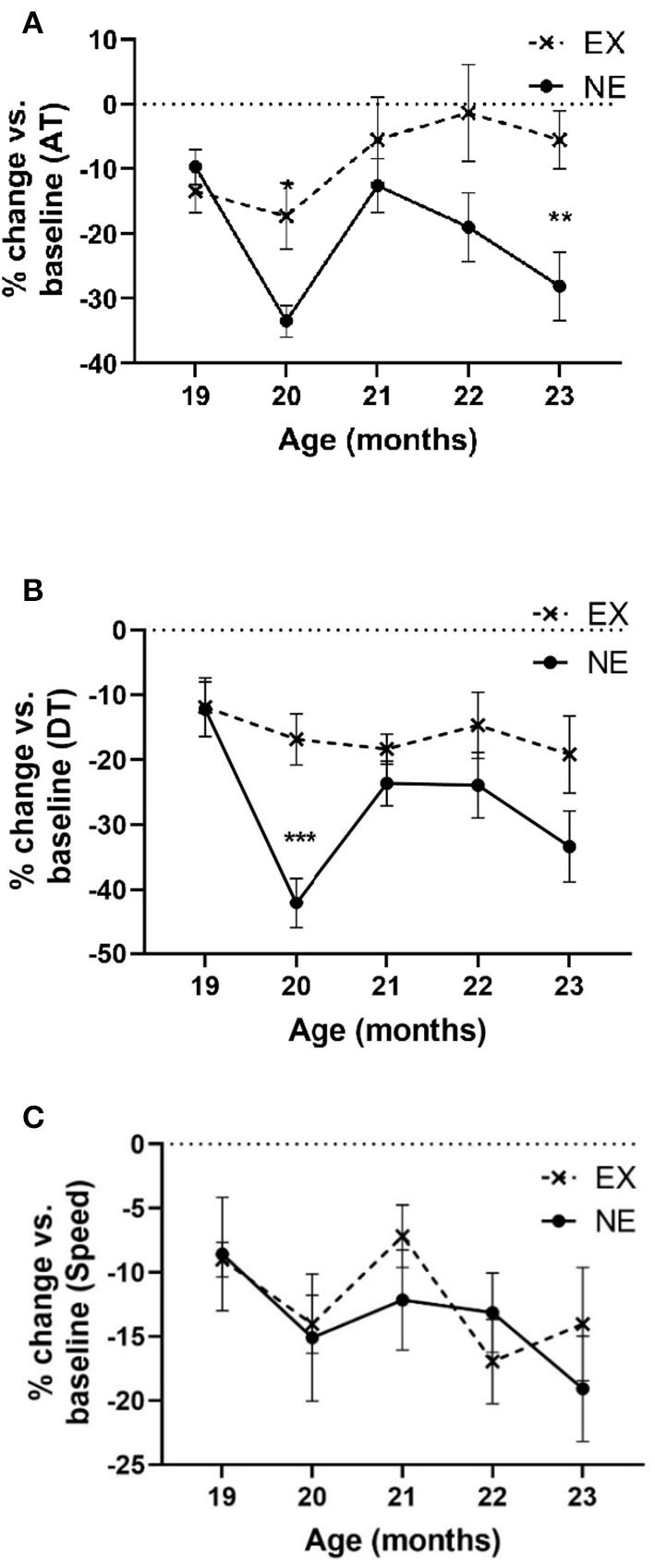
Study II. **(A)** Ambulatory time (AT) per hour. Analyses of open-field results showed significant effects of the exercise regimen on preventing aging-related decreases in AT (percentage of baseline). There was significant interaction of age × exercise [*F*_(4,51)_ = 3.10, *p* = 0.023], significant exercise effect [*F*_(1,15)_ = 6.53, *p* = 0.022], and age [*F*_(4,51)_ = 4.96, *p* = 0.002]. *Post-hoc* unpaired *t*-test revealed significant differences at 20 months (*t* = 3.01, ^*^p = 0.01, df = 13) and 23 months (*t* = 3.04, ^**^p = 0.009). **(B)** Total distance. Analyses of open-field results showed significant effects of the exercise regimen on preventing aging-related decreases in DT (percentage of baseline). No significant interaction of age × exercise was observed [*F*_(4,50)_ = 2.23, *p* = 0.08], significant exercise effect [*F*_(1,15)_ = 7.5, *p* = 0.015], and age [*F*_(4,50)_ = 5.02, *p* = 0.002]. *Post-hoc* unpaired *t*-test revealed significant differences at 20 months (*t* = 4.52, ^***^p = 0.0007, df = 12). **(C)** Speed. Analyses of open-field results showed no significant effect of the exercise regimen in speed (percentage of baseline). No significant interaction of age × exercise was observed [*F*_(4,58)_ = 0.65, ns], nor exercise effect [*F*_(1,15)_ = 0.08, ns]. Age affected speed [*F*_(4,58)_ = 2.52, *p* = 0.05].

In the 5-month longitudinal assessment of exercise impact (Study II), aging from 18 to 23 months produced a highly significant decrease in locomotor activity near ~30% in AT in the NE group (Figure 5A). In contrast, AT in the EX group decreased 6% of baseline (Figure 5A). As in Study I, the aging-related decrease in AT occurred at 20 months in the NE, with prevention of this decrease in the EX group (Figure 5A).

Aging produced a significant decline in DT traveled (~33% by age 23 months) in the NE group that was mitigated by exercise (~18% in the EX group at age 23 months) (Figure 5B). The percentage decrease in DT in the EX group did not decrease more than 19% of baseline at any month.

Aging decreased movement speed ~20% from the 18-month-old baseline and just reached significance by age 23 months old (Figure 5C), with no significant difference between the EX and NE groups (Figure 5C).

Thus, significant decreases in movement parameters related to movement initiation and frequency (AT and DT) were seen in the NE group at the 20- and 23-month-old time points in the lifespan and were mitigated or prevented in the EX group. Of note, at 21 and 22 months, AT and DT were not decreased in the NE group. This was unexpected, as we did not observe such results in our previous study at these time points (Arnold et al., 2017).

### Longitudinal Treadmill Exercise Impact on Body Weight

**Figure 6:**
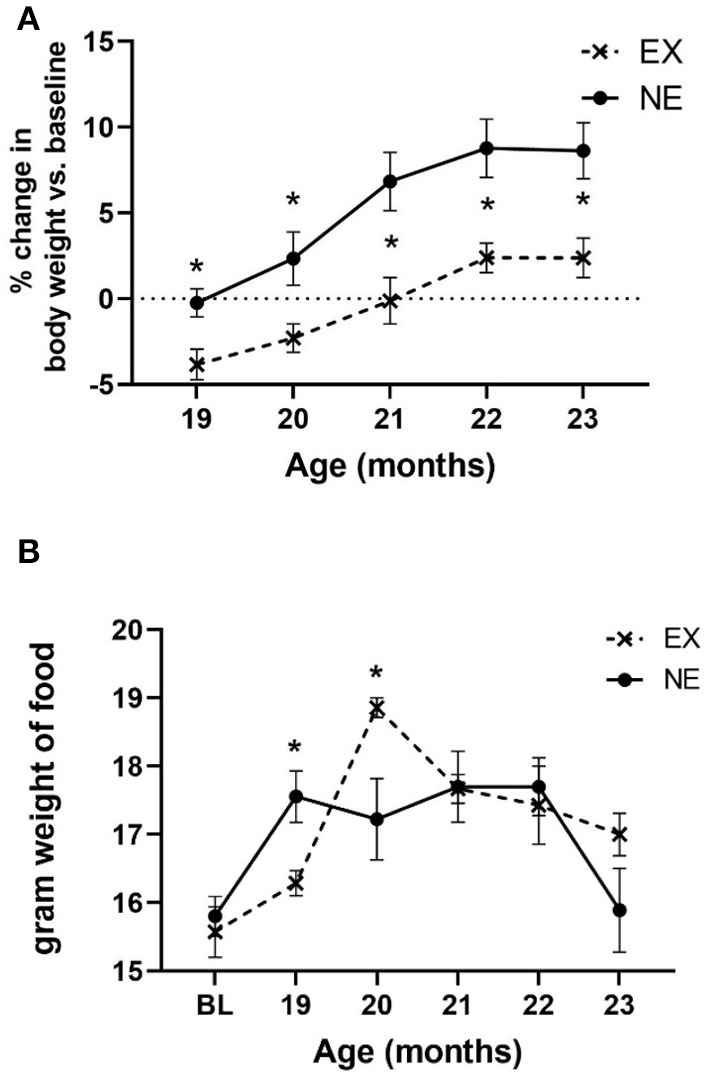
**(A)** Body weight. Body weight increased as a function of age [*F*_(4,56)_ = 26.65, *p* < 0.0001], with no significant interaction of age × exercise [*F*_(4,56)_ = 1.13, ns]. There was a significant effect of exercise on body weight gain [*F*_(1,14)_ = 9.77, *p* = 0.007], with significantly less weight recorded at each month of evaluation. Unpaired *t*-test results: 19 months (*t* = 2.85, ^*^p = 0.012, df = 14), 20 months (*t* = 2.19, ^*^p = 0.046, df = 14), 21 months (*t* = 2.87, ^*^p = 0.012, df = 14), 22 months (*t* = 2.74, ^*^p = 0.016, df = 14), and 23 months (*t* = 2.70, ^*^p = 0.017, df = 14). **(B)** Food consumption. There was a significant effect of age on food consumption [*F*_(5,71)_ = 9.34, *p* < 0.0001], and interaction of age × exercise [*F*_(5,71)_ = 3.21, ^*^p = 0.011]. Exercise effect overall was not significant [*F*_(1,15)_ = 0.27, ns]. Significant differences were observed at 19 months (*t* = 2.76, ^*^p = 0.015, df = 14), and 20 months (*t* = 2.36, ^*^p = 0.033, df = 14).

During the 5 months of Study II, body weight increased as a function of age, and exercise reduced the aging-related weight gain by the first month of exercise, and each month thereafter (Figure 6A). The increase in body weight may have been partially attributable to an increase in food consumption with age. Moreover, there was a significant interaction between age and exercise, with the EX group consuming nearly 12% more chow (by weight) (Figure 6B). However, there was no significant relationship between food consumption and locomotor performance either at 20 months of age or study conclusion (Supplementary Tables 6, 7).

There was no significant relationship between body weight against any locomotor parameters, either at baseline or study conclusion (Supplementary Tables 3, 5). Notably, there was no significant correlation between body weight and locomotor performance at 20 months of age, wherein significant mitigation of aging-related parkinsonian impairments was observed concomitant with a significant increase in food consumption (Supplementary Table 4). This lack of association of body weight with motor function has been previously reported (Arnold et al., 2017; Salvatore et al., 2017).

## Discussion

The present results demonstrate that changes in several easily measured physiological cardiovascular and body mass parameters coincide with an exercise regimen that mitigates aging-related parkinsonian signs. The increases in HR and MAP immediately following exercise were consistent throughout the regimen in the first study, with significantly greater increases in the EX group in our treadmill environment-matched and exercise-acclimated EX and NE groups. Moreover, with only 1 week of rest between exercise sessions, parkinsonian signs related to diminished movement initiation were alleviated in both studies by 20 months old. Notably, this exercise effect occurred much sooner than previously reported in our study using a regimen of identical exercise intensity and duration but with 2 weeks of consecutive rest between exercise sessions (Arnold et al., 2017). This outcome indicates rest period length, which was 2 weeks long in the aforementioned study (Arnold et al., 2017), influencing the timing of exercise efficacy to mitigate parkinsonian signs with aging. Also, with similar mitigation of parkinsonian signs by 20 months old in these two current studies (Figures 2, 5), this exercise regimen shows efficacy and reproducibility to mitigate parkinsonian signs, independent of changes in body weight (Supplementary Tables 3, 5). Moreover, the results may be translatable in humans, given the modest increase in HR and MAP associated with these motor effects. As such, this study provides predictive validity that if individuals exercise at an intensity that increases baseline or resting HR to 20–25% for at least half the days in a given month, for ~40 min per exercise day, with no more than 1 week between exercise sessions, then they may expect an alleviation of parkinsonian signs or delay its onset with consistent practice of this regimen.

The benefits of exercise on general health are widely accepted, and lifelong exercise in rats preserves aspects of motor function (Skalicky et al., 1996). However, only 59% of individuals aged 18–24 years living in the United States in 2015 met the 2008 federal physical activity guidelines (Clarke et al., 2017). With the proportion of people engaging in physical activity decreasing with age (Dawes et al., 2016), it might be assumed that a sedentary lifestyle would confer a lack of responsivity to exercise. However, our results and one previously reported (Arnold et al., 2017) indicate this may not be the case, at least into advanced middle age, as our 18-month-old rats would translate to a human age range of 45–55 years old (Quinn, 2005). However, we note the efficacy of the antiparkinsonian effects of the exercise regimen may be limited by the timing of initiation in the lifespan, as we previously reported that starting exercise 6 months later in the lifespan did not attenuate parkinsonian signs (Arnold et al., 2017). Whether this lack of exercise efficacy would still occur with a shorter rest period between exercise sessions is yet unknown, but there is evidence for improved longevity when a physically active lifestyle is initiated well into the 8th decade in the human lifespan (Stessman et al., 2009). We also note that another potential limitation of our finding is whether the alleviation of parkinsonian signs can occur with less than 5 days/week exercise frequency. This possibility will be important to follow-up since patients with early-stage PD can manage no more than three sessions per week, on average (Schenkman et al., 2018), and lifestyle issues can also hinder the practice of consistent exercise in those with parkinsonism (Afshari et al., 2017).

Our study outcomes for cardiovascular changes following exercise indicated increases in HR and MAP immediately after cessation of exercise and evidence of conditioning during the 8 week long study. First, there was a relatively low increase in VO_2_max in the first week of exercise that was unchanged at the end of the regimen at 7 weeks (Figures 3E,F). Notably, our results for the calculated VO_2_ max and HR at the selected treadmill speed of 10 m/min were very consistent with previous studies that evaluated the relationship of treadmill speed against these two measures (Wisløff et al., 2001; Høydal et al., 2007). There was an effect of weeks of regimen on the resting (pre-exercise) HR and with that a trend toward decreased HR, which has also been reported in longitudinal exercise studies (Musch et al., 1989; Wisløff et al., 2001; Høydal et al., 2007). The lack of significance in baseline HR between the EX and NE groups may be related to the necessity of gradual conditioning at a comparatively lower intensity of our regimen (estimated to be only 30% of VO_2_ max; Wisløff et al., 2001) or the possibility of a lower initial baseline HR previously reported in aged BNF rats (Hacker et al., 2006). Exercise did produce a significant decrease in baseline MAP over the course of the exercise regimen (Figures 4C,D), coinciding with the prevention of aging-related parkinsonian signs. Therefore, the cardiovascular outcomes following the exercise regimen were congruent with previous rat studies. It is also notable that the weekly amount of total exercise time of rats in this study was 175 min, which is in range of recommendations by the American Heart Association that older adults should engage in exercise at moderate intensity (defined as a noticeable increase in HR) for at least 150 min/week (Nelson et al., 2007).

A major feature of our study is ensuring that the NE group is also acclimated and exposed to a comparable protocol, with the only exception that exercise on the moving treadmill does not occur after the acclimation period. Accordingly, the NE group is subjected to the treadmill environment for the same duration and frequency as the EX group, the only difference being on a stationary treadmill. Thus, the differences in cardiovascular function between NE and EX groups were linked to the prevention of parkinsonian signs during the course of the regimen. Specifically, the moving treadmill in the EX group, and not the treadmill environment alone, produced the cardiovascular effects and attenuation of parkinsonian signs. However, it was noteworthy that exposure to the stationary treadmill also induced an increase in HR and MAP, in the NE group (Supplementary Figure 2A; Figure 3A), albeit to a significantly lesser extent than the EX group (Figures 3A,B, 4A,B). This increase in the NE group was likely due to a stress-related response that induces anxiety in anticipation for the treadmill exercise, based on previous acclimation and training experience that was implemented prior to assignment into NE or EX groups. This phenomenon has been reported by several groups by either treadmill pre-exposure paradigms (Adlam et al., 2011; Kunstetter et al., 2017) or even cage-switch stress (Morimoto et al., 2000; Adlam et al., 2011), which is an inherent component of our study as the rats must be placed back to the home cage from the treadmill to obtain the transmitter readings for HR and MAP. Despite the comparatively smaller increases in HR and MAP in the NE group immediately after exposure to the stationary treadmill, aging-related parkinsonian signs did occur in the NE group. This signifies that the movement generated in the rat by the running treadmill in the EX group that produced the modest increase (~20–25%) in HR also affected CNS mechanisms that mitigated parkinsonian signs.

Modeling parkinsonian signs in rats features the assessment of decreased locomotor activity, which is also a trait seen in human parkinsonian (Buchman et al., 2018). Our studies show that cardiovascular changes induced by a moderate treadmill exercise regimen, with an intensity and duration consistent with that which can be initiated and practiced by sedentary older adults, can attenuate parkinsonian signs in an established rat model of aging. Moreover, the results, combined with our previous observations (Arnold et al., 2017), indicate this exercise regimen attenuates parkinsonian signs much sooner (by 20 months old) by reducing the maximum rest period length to no longer than 1 week/month between exercise days. The results also show that additional potential health benefits are plausible, by evidence of reduced baseline blood pressure and modest weight loss with this exercise regimen. These cardiovascular changes are consistent with exercise training results shown in other rodent and human studies (Wisløff et al., 2001; Høydal et al., 2007; Nelson et al., 2007; Stessman et al., 2009). In addition, the improved motor parameters in the EX groups showed no correlation to weight or food consumption. Our study does not include insight into neurobiological mechanisms mediating these antiparkinsonian effects. However, there is previous evidence that the intensity and duration of this regimen increase tyrosine hydroxylase and GDNF family receptor (GFR-α1) expression in the substantia nigra (Arnold and Salvatore, 2016). To the best of our knowledge, this is the first study that employs two independent cohorts of aging rats to demonstrate exercise-induced antiparkinsonian effects occurring within the same time frame. Moving forward, these results establish a baseline of metrics upon which to evaluate the impact of exercise intensity, duration, and frequency on CNS mechanisms. Moreover, the results provide a platform to determine if there is an optimal variable of exercise to maximize its efficacy to prevent aging-related parkinsonism, particularly if initiated in sedentary older adults in the last quarter of the lifespan. For example, it is plausible that alleviation of parkinsonian signs could occur sooner, or have a greater impact if exercise intensity was increased, and to know what the accompanying increase in HR would be needed to produce such effects. As motor impairment is the most common aging-related disability and given the prevalence of parkinsonism, these results provide several angles of attack upon which to combat this major health issue and preserve functional independence in the latter half of the lifespan.

## Data Availability Statement

The original contributions presented in the study are included in the article/Supplementary Material, further inquiries can be directed to the corresponding author/s.

## Ethics Statement

The animal study was reviewed and approved by University of North Texas Health Science Center IACUC.

## Author Contributions

MS, JC, and NB obtained funding for experiments. EK and MS wrote the manuscript. EK, MS, JC, and NB edited the manuscript. EK, JL, and TM conducted experiments. EK, MS, JC, JL, and TM analyzed data. All authors contributed to the article and approved the submitted version.

## Funding

This work was supported by the National Institute on Aging (NIH grant AG040261) to MS and intramural grant funding to MS, JC, and NB by the Institute for Healthy Aging at the University of North Texas Health Science Center. EK was supported by the Office of Vice President for Research and Innovation, the Institute for Healthy Aging, and the National Institutes of Health/National Institute on Aging (T32 AG020494).

## Conflict of Interest

The authors declare that the research was conducted in the absence of any commercial or financial relationships that could be construed as a potential conflict of interest.

## Publisher's Note

All claims expressed in this article are solely those of the authors and do not necessarily represent those of their affiliated organizations, or those of the publisher, the editors and the reviewers. Any product that may be evaluated in this article, or claim that may be made by its manufacturer, is not guaranteed or endorsed by the publisher.
