# Supplementary material for: Cardiovascular Metrics Associated With Prevention of Aging-Related Parkinsonian Signs Following Exercise Intervention in Sedentary Older Rats
Source: Front Aging Neurosci. 2021 Dec 15;13:775355. doi: 10.3389/fnagi.2021.775355 (PMC8714671; doi:10.3389/fnagi.2021.775355)
Supplement: Supplementary file 1 [file Data_Sheet_1.pdf]

eTable 1 Relationship between heart rate and locomotor performance at study conclusion

	Non-Exercise		Exercise	
Locomotor Parameter	Pearson's Coefficient	*p*-value	Pearson's Coefficient	*p*-value
Ambulatory Time	-0.2436	ns	0.4229	ns
Total Distance	-0.2860	ns	0.6006	ns
Speed	-0.3423	ns	-0.3804	ns

Pearson's correlation was used to determine if a significant relationship existed between heart rate and individual locomotor performance after two months of the exercise regimen (20 months of age) for the non-exercise (NE) and exercise (EX) groups; ns = nonsignificant.

eTable 2 Relationship between mean arterial pressure and locomotor performance at study conclusion

	Non-Exercise		Exercise	
Locomotor Parameter	Pearson's Coefficient	*p*-value	Pearson's Coefficient	*p*-value
Ambulatory Time	0.1841	ns	0.0355	ns
Total Distance	0.2228	ns	-0.0100	ns
Speed	0.2480	ns	0.6926	ns

Pearson's correlation was used to determine if a significant relationship existed between mean arterial pressure and individual locomotor performance after two months of the exercise regimen (20 months of age) for the NE and EX groups; ns = nonsignificant.

eTable 3 Relationship between body weight and locomotor performance at study initiation

Locomotor Parameter	Pearson's Coefficient	*p*-value
Ambulatory Time	-0.1202	ns
Total Distance	-0.1511	ns
Speed	-0.2515	ns

Pearson's correlation was used to determine if a significant relationship existed between body weight and individual locomotor performance at study initiation for the longitudinal exercise study; ns = nonsignificant.

eTable 4 Relationship between body weight and locomotor performance at 20 months of age

	Non-Exercise		Exercise	
Locomotor Parameter	Pearson's Coefficient	*p*-value	Pearson's Coefficient	*p*-value
Ambulatory Time	0.0060	ns	-0.3783	ns
Total Distance	-0.2238	ns	-0.5649	ns
Speed	-0.3411	ns	-0.4306	ns

Pearson's correlation was used to determine if a significant relationship existed between body weight and individual locomotor performance after two months of the exercise regimen (20 months of age) for the NE and EX groups in the longitudinal exercise study; ns = nonsignificant.

eTable 5 Relationship between body weight and locomotor performance at 23 months of age

	Non-Exercise		Exercise	
Locomotor Parameter	Pearson's Coefficient	*p*-value	Pearson's Coefficient	*p*-value
Ambulatory Time	0.0803	ns	0.2251	ns
Total Distance	-0.0822	ns	-0.5640	ns
Speed	0.2277	ns	0.3791	ns

Pearson's correlation was used to determine if a significant relationship existed between body weight and individual locomotor performance at study conclusion for the NE and EX groups for the longitudinal exercise study; ns = nonsignificant.

eTable 6 Relationship between food consumption and locomotor performance at 20 months of age

	Non-Exercise		Exercise	
Locomotor Parameter	Pearson's Coefficient	*p*-value	Pearson's Coefficient	*p*-value
Ambulatory Time	0.3348	ns	-0.1811	ns
Total Distance	0.2806	ns	-0.6461	ns
Speed	0.1453	ns	-0.3574	ns

Pearson's correlation was used to determine if a significant relationship existed between food consumption and individual locomotor performance after two months of the exercise regimen (20 months of age) for the NE and EX groups in the longitudinal exercise study; ns = nonsignificant.

eTable 7 Relationship between food consumption and locomotor performance at 23 months of age

	Non-Exercise		Exercise	
Locomotor Parameter	Pearson's Coefficient	*p*-value	Pearson's Coefficient	*p*-value
Ambulatory Time	-0.0065	ns	0.3069	ns
Total Distance	0.0916	ns	0.7024	ns
Speed	0.1991	ns	0.4695	ns

Pearson's correlation was used to determine if a significant relationship existed between food consumption and individual locomotor performance at study conclusion for the NE and EX groups in the longitudinal exercise study; ns = nonsignificant.

eFigure 1.

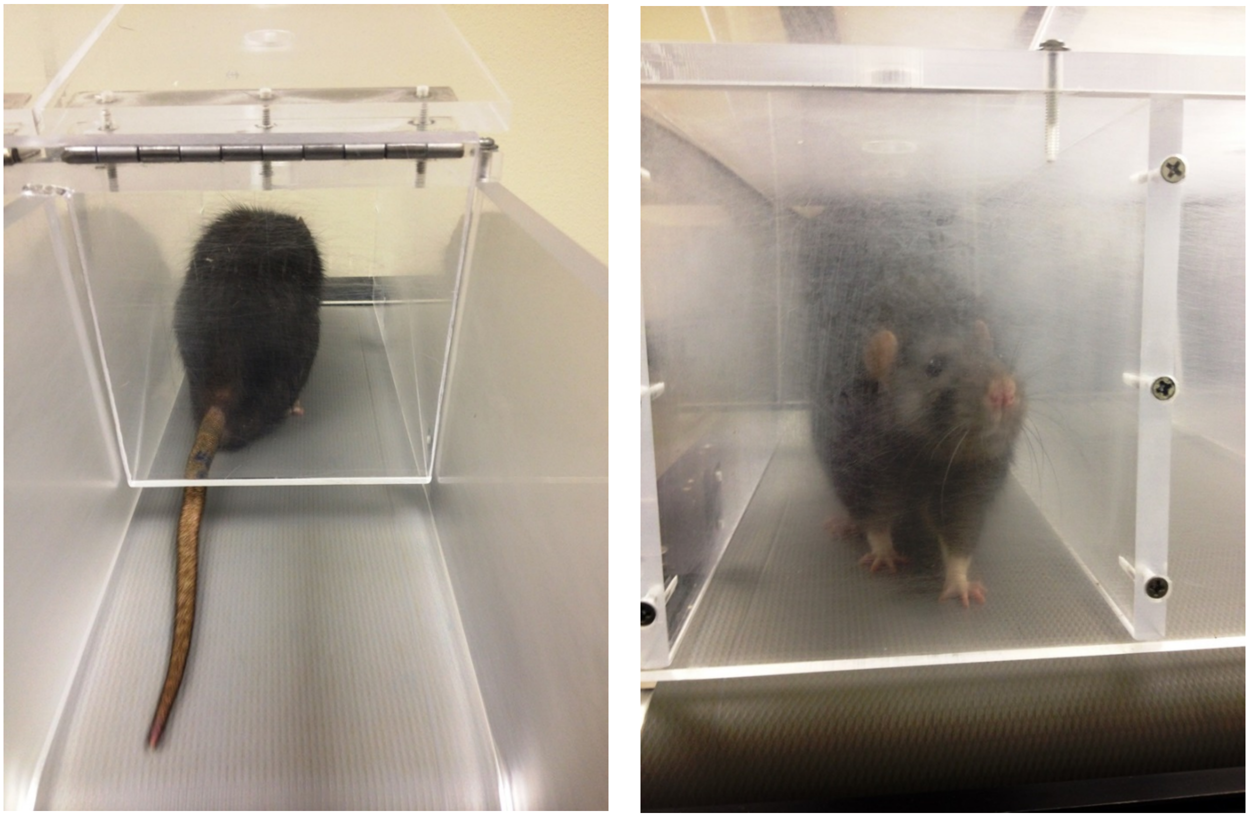

Treadmill exercise environment. The Brown-Norway F_1 hybrid rat is accommodated to comply to the footshock-free exercise regimen. In the left image, the clear plexiglass backstop is used to motivate the rat to keep moving, as necessary to maintain compliance, on the running treadmill. In the right image, the rat is maintaining exercise compliance by keeping pace near the front end of the treadmill.

eFigure 2.

A. **B.**

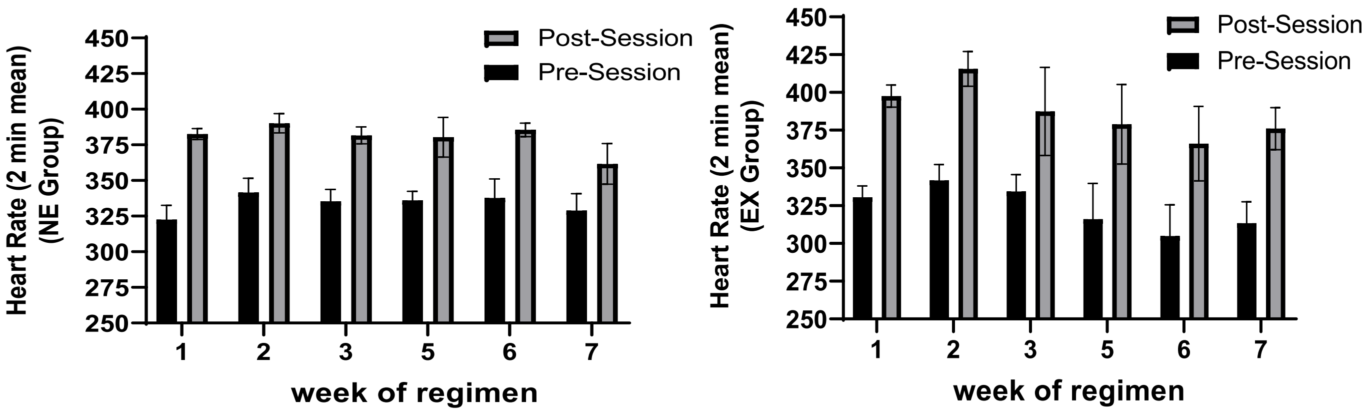

Treadmill environment impact on heart rate (HR). A. Stationary treadmill (NE Group). Exposure to the stationary treadmill for the same duration as the EX group increased HR above pre-exposure levels ($F_{(1,7)}$=40.9, p=0.0004) with no effect of weeks of regimen ($F_{(5,35)}$=1.12, ns) or weeks of regimen x exposure to treadmill interaction ($F_{(5,35)}$=0.95, ns). **B. Running treadmill (EX Group).** There was a significant effect of exercise ($F_{(1,6)}$=459.4, p<0.0001) but no significant effect of weeks of regimen ($F_{(5, 30)}$=1.91, ns) or weeks of intervention x exercise interaction ($F_{(5,30)}$=0.25, ns).

eFigure 3.

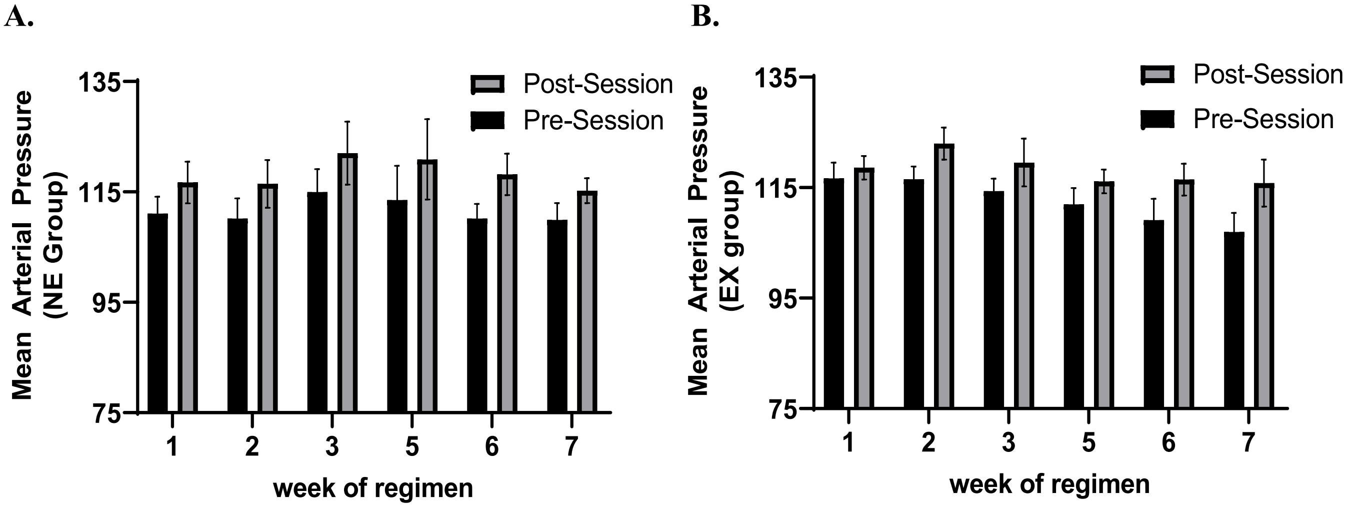

Treadmill environment impact on mean arterial pressure (MAP). A. Stationary treadmill (NE Group). Exposure to the stationary treadmill for the same duration as the EX group increased MAP above pre-exposure levels ($F_{(1,7)}$=19.35, p=0.003), with no effect of weeks of intervention ($F_{(5,35)}$=0.72, ns) or weeks of intervention x exposure to treadmill interaction ($F_{(5,33)}$=0.34, ns). **B. Running treadmill (EX Group).** The exercise regimen increased MAP ($F_{(1,6)}$=24.56, p=0.003), with no significant effect of weeks of intervention ($F_{(5, 30)}$=1.92, ns) or weeks of intervention x exercise interaction ($F_{(5,29)}$=2.08, p=0.097).
